# Particles in Band Saw Coolant: Size Distributions and Implications for Guide Clearances and Friction

**DOI:** 10.3390/ma19030555

**Published:** 2026-01-30

**Authors:** Matthias Schmid, Tobias Tandler, Hans-Christian Möhring, Katharina Schmitz

**Affiliations:** 1 Institute for Fluid Power Drives and Systems, RWTH Aachen University, 52074 Aachen, Germany; katharina.schmitz@ifas.rwth-aachen.de; 2 Institute for Machine Tools, University of Stuttgart, 70174 Stuttgart, Germany; tobias.tandler@ifw.uni-stuttgart.de (T.T.); hans-christian.moehring@ifw.uni-stuttgart.de (H.-C.M.)

**Keywords:** metalworking fluid (MWFs), contamination, cutting, friction

## Abstract

In metal band sawing, higher cutting speeds increase frictional heat at sliding guide blocks. Recirculating water-miscible metalworking fluids (MWFs) often lack fine filtration and accumulate debris that can enter the guide–band interface. A 1 L coolant sample collected after 22.5 m^2^ of cutting contained a particle load of 0.438 g/L; optical sizing yielded a number-median maximum Feret diameter of 345 µm, with particles up to 1.5 mm. Compared with typical guide clearances (~0.1 mm), these sizes imply frequent ingress/bridging and three-body interactions. The coolant viscosity follows an Andrade relation and decreases by ~2% K^−1^ around 40 °C. HFRR tribometry indicates low steady-state friction (µ ≈ 0.12), comparable to cutting oil. Together, these results provide quantitative design inputs for next-generation guide clearances and targeted filtration/coolant-delivery concepts in high-speed band sawing.

## 1. Introduction

Machining processes rely heavily on cooling lubricants to manage heat and friction, thereby enhancing both tool life and workpiece quality. These fluids play a pivotal role in dissipating the heat generated during cutting, reducing friction at the tool–workpiece interface, and flushing away debris, which collectively contributes to improved machining performance and economic efficiency [1,2]. In band sawing, which is a critical initial step in many manufacturing processes, the effective use of cooling lubricants is particularly important due to the process’s unique challenges, such as low cutting speeds, band instability, and limited access for lubricants to reach the cutting zone. Despite its importance in industrial applications, research into band sawing lags behind other machining processes such as turning, milling, and drilling, leaving significant gaps in our understanding—particularly with regard to the impact of particle contamination in cooling lubricants.

Unlike most metal-cutting processes, band sawing involves a compliant band that must be stabilised by guide systems located close to the kerf. These guides not only provide geometric constraints but also act as tribological interfaces: frictional power increases with cutting speed and normal load, and the resulting heat input directly affects band straightness, residual stress, and tracking stability. Recent work shows that guide devices can even function as actuators to influence tilting of the band during the process, enabling active compensation for tool deflection [3]. This emphasises that the design of the guides and their tribological operating window are increasingly important factors in determining productivity and cut quality at higher speeds.

There is some research literature on the role of cooling lubricants in various machining processes. In turning and milling, significant progress has been made in lubrication technology. Advancements such as minimum quantity lubrication (MQL) have led to widespread adoption of this technique, with the primary objective of reducing cooling lubricant consumption while maintaining or enhancing machining performance. MQL has been shown to reduce manufacturing costs and environmental impact by using significantly less fluid than traditional flood cooling [4]. Furthermore, the incorporation of nanofluids and solid lubricants, such as molybdenum disulfide (MoS_2_) mixed with vegetable oils, has been demonstrated to enhance heat transfer and reduce friction, leading to improved tool life and surface quality [5,6].

In related abrasive machining processes (e.g., grinding), fine particles in the low-micron range can contaminate process fluids, so advanced purification such as belt/paper filtration and/or centrifugation is used to prevent recirculation of fines and to maintain process quality [7].

Supplying coolant directly into the guide–band interface is an attractive route to reduce friction and guide heating and, in fluid-pocket concepts, to generate a load-carrying film that increases damping and suppresses vibration. In stone band sawing, a comparable approach has been demonstrated using hydrostatic guides with nozzle arrays integrated into the guide element, where pressurised fluid jets align the blade and reduce passive forces [8]. Although stone sawing operates with water rather than metalworking emulsions, the principle of contactless guidance through fluid pressure offers a potential pathway for metal band sawing—provided that coolant cleanliness can be assured.

Transferring such concepts to metal band sawing requires explicit consideration of coolant cleanliness: most industrial metal band saws operate with coarse filtration and recirculation, so metallic debris can enter or bridge guide clearances and convert a two-body sliding contact into a particle-mediated three-body contact, degrading both friction and wear behaviour. Three-body lubrication modelling shows that decreasing viscosity—whether due to temperature rise or fluid degradation—makes particle-contaminated interfaces more prone to enter boundary lubrication and suffer surface damage compared with clean two-body contacts [9]. This coupling is particularly relevant in guide jaws, where small clearances and high sliding speeds amplify both viscous heating and particle entrainment.

Field data indicate that metalworking fluids can accumulate a substantial particulate burden under production conditions. In an industrial band saw environment, microparticles in used MWFs have been characterised and linked to fluid degradation mechanisms; the reported particle populations include wear debris and oxide-containing particles, highlighting that guide and pump circuits can act as continuous contamination sources during long running times [10]. Beyond particulate contamination, microbial loads (including pathogenic strains) have also been documented, underscoring the need for comprehensive fluid management strategies.

The problem of restricted kerf access is not unique to band sawing. In circular sawing, studies have investigated the effectiveness of the internal cooling lubricant supply; however, the feed rates in circular sawing are significantly higher than in band sawing, so the relative impact of contamination may differ [11]. Compared to other machining processes involving geometrically defined cutting edges, such as milling or turning, band sawing is characterised by a significantly lower feed per tooth. Sarwar et al. [12] reported that the feed per tooth in band sawing typically ranges from 5 to 30 µm, reflecting the process’s reliance on a large number of cutting edges with minimal material removal per pass. This low feed rate affects the size and distribution of particles in the cooling lubricant, as smaller chips may produce finer particles that are more difficult to filter out.

The cooling lubricant systems employed in band sawing machines are frequently engineered to be cost-effective, resulting in the utilisation of rudimentary filtration solutions in lieu of dedicated filtration systems. Consequently, the presence of small particles, such as metal chips or abrasive debris, is a persistent issue. These particles remain in the lubricant and are recirculated through the system via pumps, with the potential to reach the cutting zone and band guides. This recirculation can exacerbate wear on the saw band and machine components, thereby compromising both cutting efficiency and workpiece surface quality.

Wolters et al. [13] examined the use of various cooling methods for cutting 3D-printed parts, comparing MQL, flood cooling, and air cooling. The study highlights the influence of these strategies on heat dissipation, friction, and particle transport in the sawing process, demonstrating that MQL shows potential for reducing lubricant consumption while maintaining effective cooling and chip evacuation—findings that may inform future band saw coolant delivery concepts.

Beyond chemical formulation, the tribological performance of water-miscible MWFs is governed by temperature-dependent rheology and by the presence of solid third bodies. Recent comprehensive studies on cutting oils and water-based MWFs have characterised their rheological, tribological, and thermal behaviour, showing that water-based fluids exhibit significant shear thinning while cutting oils maintain nearly Newtonian properties [14]. Complementary tribology studies highlight that many commercial water-miscible MWFs primarily operate in boundary lubrication rather than forming stable hydrodynamic or elastohydrodynamic (EHL) films over wide speed ranges, which has direct implications for guide contacts [15,16]. Work on lubricated cutting also emphasises limited MWF penetration and the dominance of boundary lubrication in highly loaded, fast-sliding interfaces [15].

Accordingly, coolant delivery concepts for guide-integrated lubrication must be paired with cleanliness management. Recent studies demonstrate that advanced treatment routes—such as nanofiber filtration combined with ozone—can reduce both microparticles and microorganisms in process fluids [17]. In parallel, sensor-based monitoring concepts for cutting emulsions and standardised approaches for particulate contamination monitoring provide building blocks for condition-based maintenance and for defining cleanliness targets matched to guide clearances.

The present study addresses these knowledge gaps by quantifying the in-operation particulate load and its consequences for guide–band interaction. Coolant sampled after extended cutting is analysed for particle size distribution and compared with typical guide-jaw clearances. The coolant’s temperature-dependent viscosity is characterised, and its frictional behaviour is classified using standardised tribometry. The combined evidence provides a basis for targeted countermeasures in coolant delivery and filtration and for the design and operation of sliding guide systems under high-speed cutting conditions.

## 2. Materials and Methods

### 2.1. Setup

**Figure 1:**
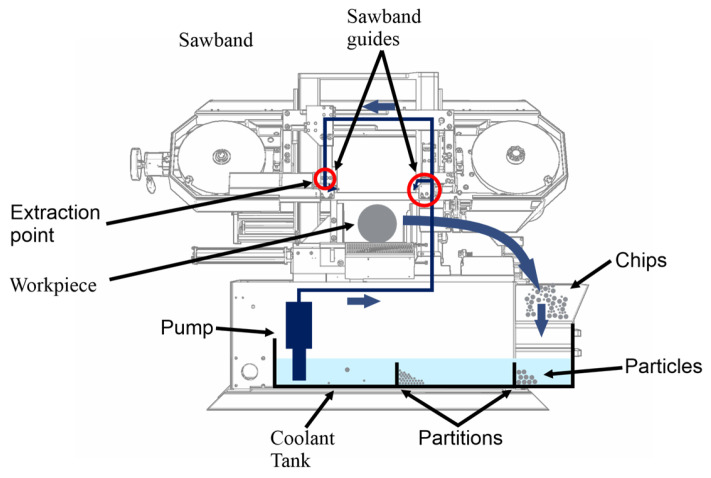
Flow path of the coolant in the Klaeger Pharos 300 (Kernen im Remstal, Germany) and basic components.

The saw’s cooling lubricant tank has several partitions. Cooling lubricant enters the tank on the right-hand side. It then passes through the chip box of the machine. The cooling lubricant pump, which is a simple submersible pump without a filter, is mounted on the left-hand side of the tank. This structure is shown schematically in Figure 1.

The coolant sample was taken at the end of a series of tool-life tests, after a cumulative cutting area of 22.5 m^2^ had been achieved. This sampling point was deliberately chosen to capture a mature contamination state representative of extended industrial operation. Given that the test campaign extended over several weeks, the coolant had sufficient time to reach a quasi-steady state in terms of particle burden, balancing continuous debris input from cutting against sedimentation in the tank partitions. For designing guide clearances and filtration systems, this near-worst-case scenario is the most relevant, as it defines the upper limit of particle sizes and concentrations that the guide system must tolerate.

One limitation of this study is that the temporal evolution of particle concentration during the cutting campaign was not tracked. Future work should characterise contamination build-up from fresh coolant in order to identify critical thresholds and optimal maintenance intervals.

HSS band saw bands were used to make cuts at a cutting speed $v_{c\;}$ = 53 m/min and a feed rate of $f_{f}$ = 26 mm/min. These parameters are recommended by the saw band manufacturer for the material. The cooling lubricant is an emulsion “Avilub Metacool BFH” manufactured by Bantleon (Ulm, Germany). The parameters are shown again in the Table 1 for ease of reference.

The performance of the cooling lubricant in this setup is governed by its physical properties, which are essential to understanding how it behaves during the sawing process. These properties are discussed in the following section.

### 2.2. Fluid Properties

To physically describe and influence the sawing process, various key figures are required. One example is the Reynolds number, which represents the relationship between inertial and viscous forces on the fluid. It is determined using Equation (1).

(1) $$Re=\;\frac{\rho \;\cdot u\;\cdot d}{\eta }$$

In this case, the velocity and the characteristic length are determined by the sawing process. However, the density and viscosity of the cooling lubricant remain significant factors. Additionally, the contact area acts as a tribological system comprising two friction partners. The band saw band moves relative to the cutting material, causing the fluid to move in the gap. The friction that occurs here is determined by the lubricating effect of the fluid, among other things. A coefficient of friction can be determined for classification purposes.

The methods for recording the fluid parameters are presented below. Since these are sensitive measuring instruments, the sawn chips must be separated from the cooling lubricant. This is the only way to ensure that the measuring instruments are not damaged. In addition, the particles themselves must also be geometrically measured. They are therefore separated using filter paper, then cleaned with isopropanol and dried again. The particle sizes are then measured optically under a microscope (Alicona; 50x-Zoom, Itasca, IL, USA).

#### 2.2.1. Viscosity

A Ubbelohde viscometer in accordance with DIN 51562 is used to determine the kinematic viscosity [18]. The temperature of both the sample cooling lubricant and the viscometer itself is controlled with an accuracy of 0.01 K. Homogeneous temperature conditions are ensured for each measurement by preconditioning the entire viscometer in a temperature control unit for half an hour beforehand. The measurement process is repeated five times to ensure reproducibility [19].

**Figure 2:**
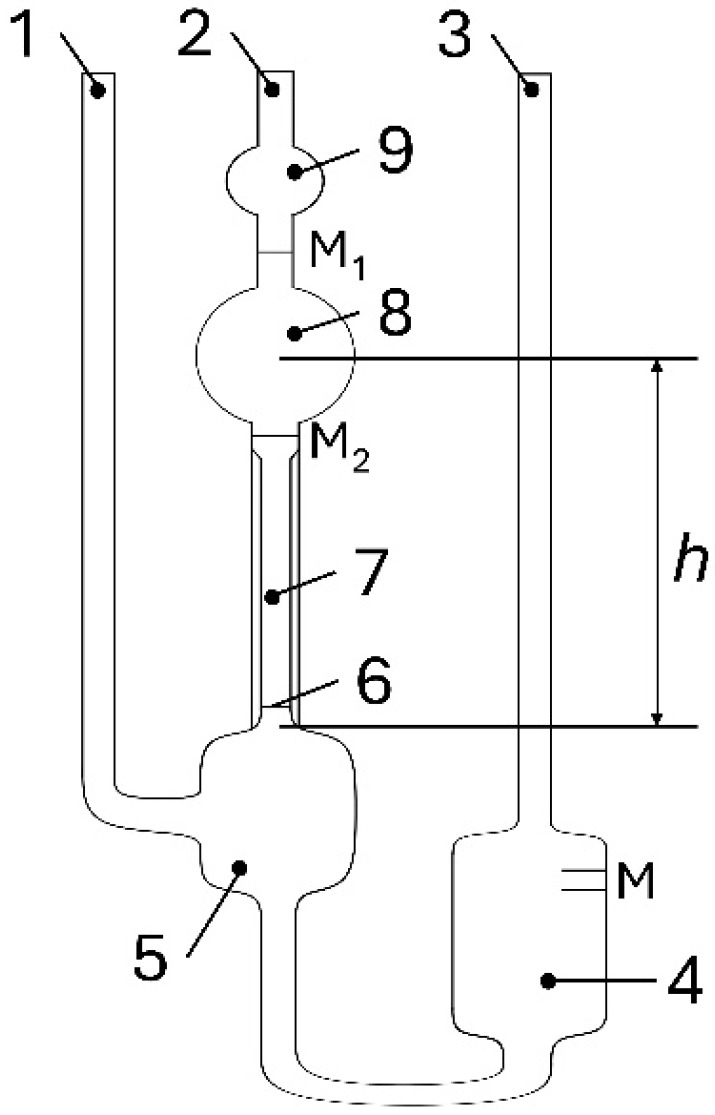
Ubbelohde viscometer [18].

The test setup is shown in Figure 2. The device consists of three parallel tubes. A storage vessel is attached to tube 3. The flow ball (9) and the measuring vessel (8) are located under tube 2, to which a capillary (7) is connected; (5) indicates the level of the vessel. The ring marks M_1_ and M_2_ are used to define the flow volume of the sample and the mean pressure head h.

The liquid sample to be characterised is filled through tube 3 into the storage vessel (4) until the fill level reaches between the markings (M). The device is located in a temperature-controlled bath. Once the test temperature has been reached, tube 1 is closed and suction pressure is applied to tube 2. This fills the level vessel (5), the capillary (7), the measuring vessel (8), and the lead ball (9) in succession. The suction pressure is then removed and the opening on tube 1 is reopened. This causes the liquid to break off at the lower end of the capillary (7). The so-called “hanging ball level” forms in the ball cup (6) below. The decisive measurement parameter is the time span within which the lower edge of the meniscus of the sample descends from the upper edge of the ring measurement mark M_1_ to the upper edge of the ring measurement mark M_2_.

Depending on the capillaries used, a correction factor is specified as the device constant K [mm^2^s^−2^]. When supplemented by the measured flow time t [s], the kinematic viscosity ν can be calculated according to Equation (2).

(2) ν= K ·t

#### 2.2.2. Density

**Figure 3:**
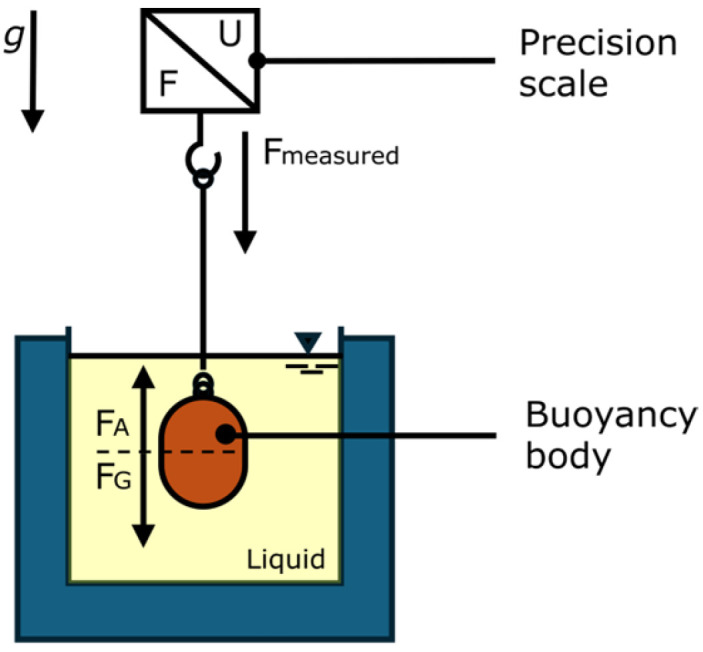
Test setup for density measurement [20].

Figure 3 shows the setup used to determine the density of the cooling lubricant. A buoyancy body is immersed in the liquid. This is connected to a precision balance. The balance measures the weight force minus the buoyancy force. Given the known volume and mass of the buoyant body, the density of the liquid can be calculated according to Archimedes’ principle. This is performed using Equation (3) [20].

(3) $$\rho_{liquid}=\frac{m_{BB}-m_{measured}}{V_{BB}}$$

**Figure 4:**
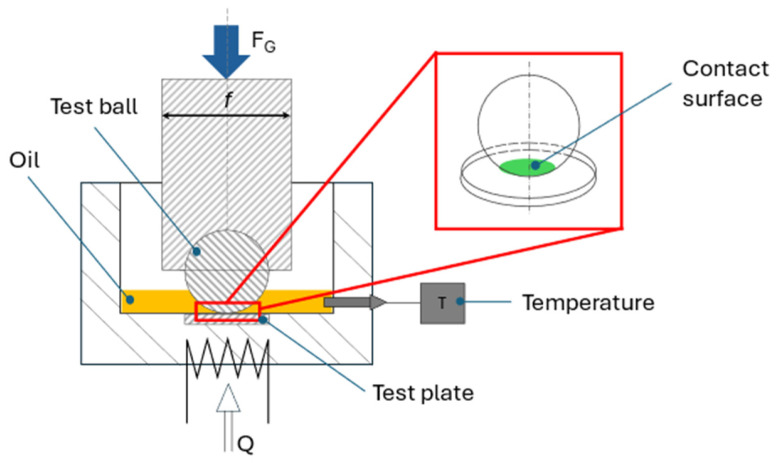
Setup for HFRR test [21].

In addition to the flow properties within the gap, the resulting tribological properties are also important. In the saw kerf itself, the friction surfaces (the saw band and the cutting material) are wetted by the cooling lubricant. This completes the tribological system [21]. The system is classified using the HFRR test in accordance with DIN EN ISO 12156-1 [22] to assess the lubrication properties. This enables the coefficient of friction to be determined. For this purpose, a ball made of steel ISO 683-17-100Cr6 [23] mounted in a holder is pressed onto a polished steel disc with a defined force, as shown in Figure 4. The maximum Hertzian pressure can be calculated according to Equation (4). With the material-dependent characteristics, Poisson’s ratio v and modulus of elasticity E [24], and an applied force $F_{G}$ of 2.04 N, this results in a maximum Hertzian pressure of 835.68 N/mm^2^ [25]. Although this does not reflect the contact pressure in the saw gap in detail, it allows for comparability with equivalent measurements and therefore suggests analogous effects in similar processes.

(4) $$p_{max}=\left(\frac{1}{\pi }\right)\;\cdot {\left\{\frac{1.5\;\cdot \;F_{G}}{r^{2}}\cdot {\left(\frac{E}{1-v^{2}}\right)}^{2}\right\}}^{1/3}$$

The disc is placed in a container filled with the cooling lubricant. During the 75 min test procedure, the temperature of the liquid is regulated to 60 °C. The steel ball oscillates at a frequency of 50 Hz with a stroke of 1 mm on the polished steel disc. Due to the cooling lubricant’s inhomogeneous composition, caused by chips and varying emulsifier content, this process is repeated three times [20,26].

## 3. Results

This section presents the results of the measurement methods used to characterise the cooling lubricant in terms of its particle load, viscosity, and friction behaviour. Additional measurements were carried out with water, in dry running conditions, and with standard cutting oil to facilitate comparison and categorization. The results offer valuable insights into the performance of the metalworking fluid in the band sawing process, as well as its impact on particle contamination and system design.

### 3.1. Particle Load

**Figure 5:**
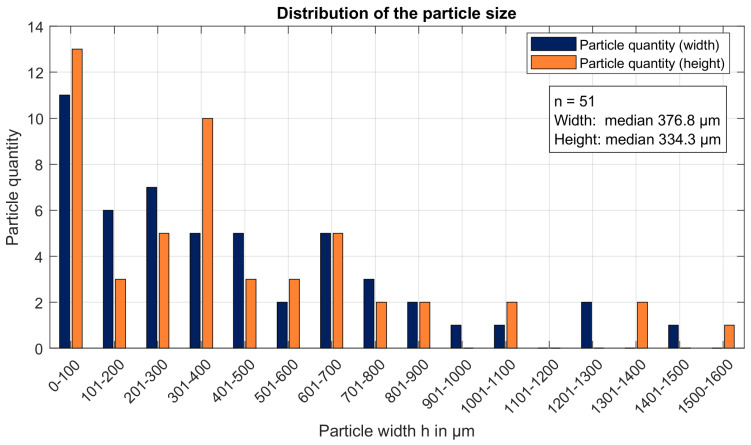
Number-based particle size distribution in a 1 L sample of recirculating coolant after 22.5 m^2^ of cutting. Median maximum Feret = 345 µm.

Figure 5 illustrates the particle size distribution in one litre of cooling lubricant following a 22.5 m^2^ cutting area. These particle sizes are important for determining an appropriate gap height in the sawing system, as they directly affect friction due to particle entrainment. The particles were separated from the cooling lubricant using a paper microfilter and dried after cleaning with isopropanol. This resulted in a total particle load of 0.438 g/L. As can be seen from the distribution, most particles are concentrated in the smaller size range of 100–400 µm. More precisely, the 50th percentile limit is 345 µm based on the largest particle size. This is attributed to the thin, unstable structure of the sawdust, which probably fragments further during circulation in the system. The reduction in larger particles is also due to intermediate filtration using a wire mesh, which retains coarse impurities while allowing finer particles to pass through.

**Figure 6:**
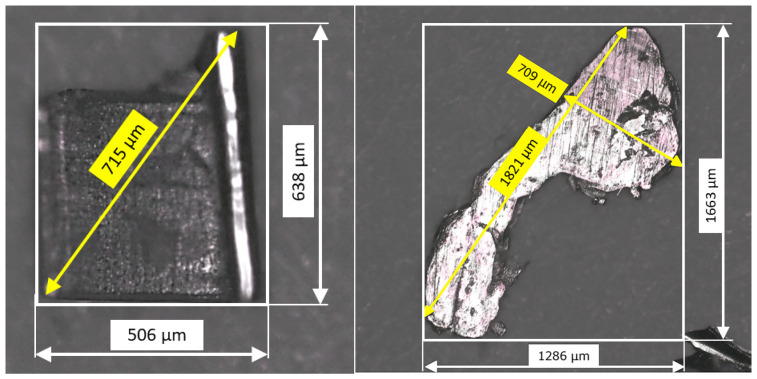
Exemplary measurement (Alicona; 50x-Zoom) of the particle size.

The particle size was determined by measuring the maximum extension of each particle, defined as the longest distance within the particle (see Figure 6) [27]. This parameter is important because the particles can orient themselves to fit through the slit perpendicular to their longest axis. Therefore, the maximum extension serves as the basis for estimating the required gap height to minimise friction. Additionally, the orthogonal width of the particles was measured to determine the appropriate filter fineness, ensuring efficient particle removal while maintaining adequate lubricant flow. This method can only be considered an estimate, as particle alignment in the actual flow in the gap cannot be recorded.

### 3.2. Viscosity Change Behaviour

**Figure 7:**
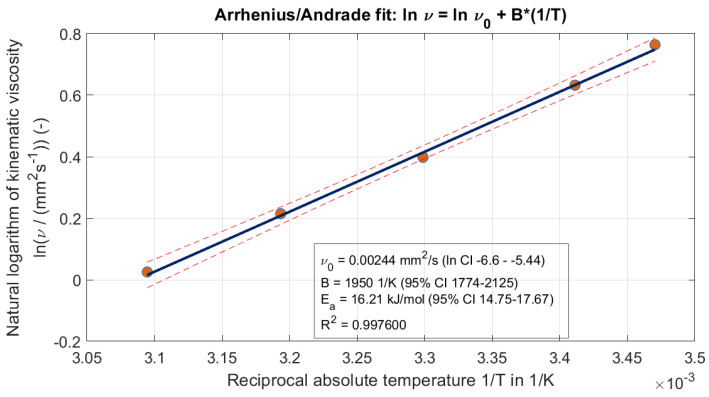
Arrhenius/Andrade representation of the kinematic viscosity.

**Figure 8:**
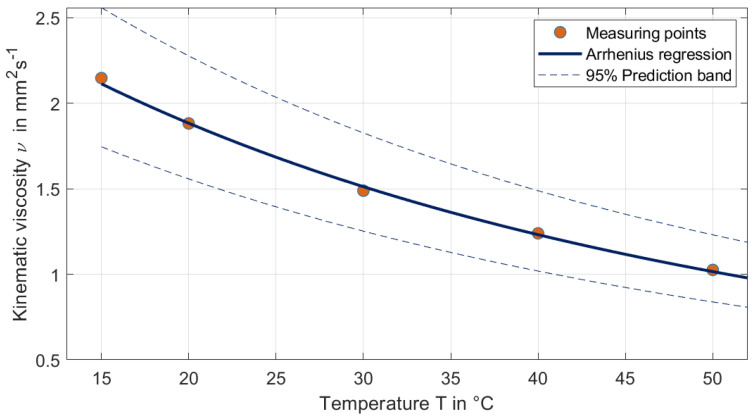
Temperature dependence of kinematic viscosity ν(T) with 95% prediction band.

The kinematic viscosity of the filtered cooling lubricant was measured at discrete temperatures and approximated using an Arrhenius regression model (Figure 7). Each temperature point was measured five times to ensure reproducibility. Figure 8 demonstrates the quality of this approximation, showing a clear decrease in viscosity with increasing temperature. The measurements were conducted over a temperature range relevant to typical operating conditions. This temperature-dependent behaviour has significant implications for the design of the guide gap in the band saw band, as reduced viscosity alters the flow properties and the lubricant’s ability to maintain a stable film.

Around 40 °C, the kinematic viscosity decreases by ~2% per °C ($Q_{10}\approx 1.21$), i.e., a 20 °C increase (40 → 60 °C) reduces ν by ~32%. Consequently, the dimensionless film parameter (and thus the Stribeck number) scales down by the same factor, promoting mixed/boundary lubrication and increasing sensitivity to particulate entrainment. Using the fit, we obtain $\nu (60\;{}^{\circ}C)=0.847\;{\mathrm{mm}}^{2}s^{-1}$ with a 95% prediction interval of 0.697–1.031 mm^2^s^−1^.

### 3.3. Frictional Behaviour

**Figure 9:**
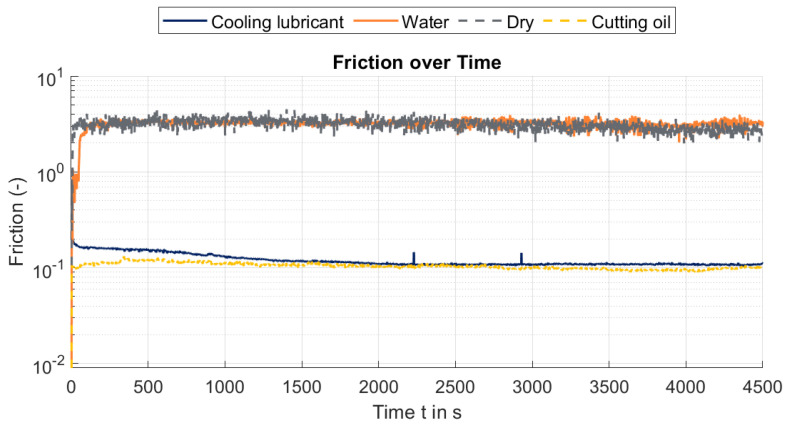
Friction over time of cooling lubricant, water, dry, and cutting oil conditions.

The frictional behaviour of the cooling lubricant was investigated using the High-Frequency Reciprocating Rig (HFRR) test in accordance with DIN EN ISO 12156-1. Figure 9 presents the time-dependent evolution of the friction coefficients for the cooling lubricant compared to reference measurements with water, dry operation, and a standard cutting oil, plotted on a logarithmic scale. The analysed cooling lubricant exhibits frictional properties similar to those of the cutting oil, approaching an asymptotic value of 0.107, indicating stable lubrication performance.

**Figure 10:**
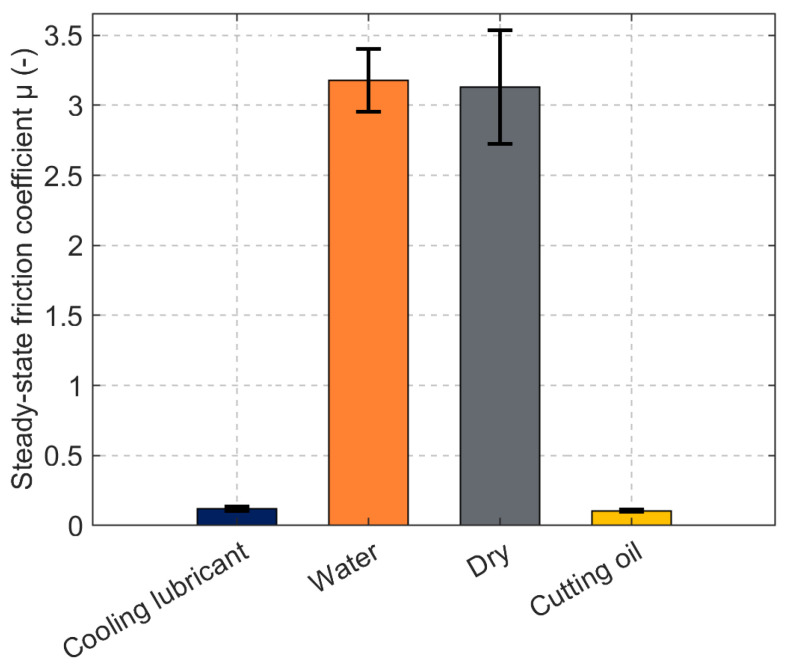
Steady-state friction of cooling lubricant, water, dry and cutting oil conditions. Error bars represent the standard deviation of the friction coefficient during steady-state conditions.

To highlight the steady state, the friction coefficient is averaged over the last 50 s of each run and presented in Figure 10 and Table 2. The error bars in Figure 10 represent the standard deviation of the friction coefficient during steady-state conditions.

During the HFRR test, the oscillating movement of the steel ball displaces the lubricant on the steel disc, leading to fluctuations in film thickness. The film percentage indicates the degree of surface separation between the oscillating ball and the test disc, measured via electrical contact resistance. A value of 100% represents complete separation by a lubricating film (full-film lubrication), while 0% indicates direct metal-to-metal contact (boundary lubrication).

**Figure 11:**
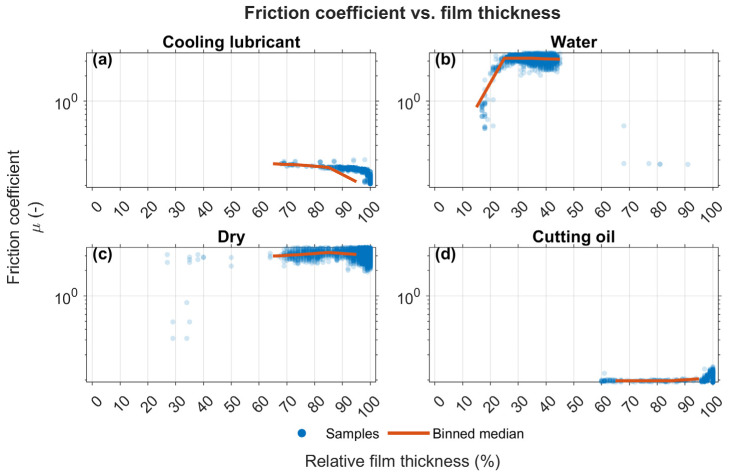
Distribution of friction coefficients according to film thickness.

This phenomenon is reflected in the noise observed in the friction coefficient measurements over time (see Figure 9 and Figure 10). Figure 11 shows the relationship between the coefficient of friction and film thickness for the tested conditions. While cutting oil maintains a relatively constant film thickness, water shows a significant decrease that correlates with higher friction coefficients approaching those of dry friction.

## 4. Discussion

The nominal guide clearance of about 0.1 mm must be evaluated against the debris actually present in the coolant. The sizing shows a number-median maximum Feret of ≈345 µm, so particles can approach or exceed the gap depending on orientation and deformability. Even when “oversized” particles squeeze through, transient bridging and three-body interactions can intermittently pry the jaws apart and excite band vibrations, so the instantaneous minimum gap—and thus ingress probability and abrasive risk—becomes time-dependent rather than purely geometric. Increasing clearance reduces the chance of bridging but weakens lateral guidance; a practical target is to choose the gap g such that the exceedance probability based on the orthogonal particle width is small. This links filter cut-off, particle statistics, and gap selection, and in practice, argues for a pre-filtration cut-off upstream of the guides. Viscosity provides the second lever. The Andrade fit indicates a strong temperature sensitivity around 40 °C, and the kinematic viscosity decreases by ≈2% per K (Q_10_ ≈ 1.21), i.e., a 20 °C increase reduces ν by ≈32%.

Because hydrodynamic film thickness and squeeze-film damping scale roughly with dynamic viscosity μ=ρν, hotter coolant shrinks both film and damping, pushing the contact toward mixed/boundary lubrication and amplifying the influence of entrained debris. Operationally, one can compensate for drifts by keeping a Stribeck-type parameter S∝μU/p approximately constant, adapting pressure or speed. While pressure does not change μ, higher supply pressure promotes flushing, helps stabilise an effective gap, and can damp vibration through squeeze-film action in small clearances.

The HFRR benchmark supports this picture: the emulsion reaches a steady-state friction coefficient of ≈0.120, comparable to cutting oil and far below water or dry operation. Thus, provided that the film is not disrupted by debris, boundary chemistry together with sufficient μ at 60 °C sustains low friction; conversely, water-like conditions (overheated or overly diluted emulsions) collapse μ and erode damping, allowing particles to dominate the contact mechanics. Nevertheless, the combined evidence translates directly into design practice: select filtration and gap based on the cumulative distribution function of orthogonal widths, keep the bath near 40–45 °C or apply the simple μ-based scaling above to hold lubrication conditions constant, and use the fitted $\nu \left(T\right)$ (and μ(T), if ρ(T) is available) to simulate guide behaviour across realistic temperature ranges.

## 5. Conclusions

This study examines the properties of the cooling lubricant (emulsion) and the loading of contaminants under real band saw operating conditions. The aim was to understand the limits of feasible band speeds and the stability of sliding band guidance. Measurements showed that the recirculating cooling lubricant could carry debris up to 1.5 mm in size, which is large enough to interfere with lubrication in the guide jaws and disrupt thin-film conditions in the guide gaps. Where such particles measurably impair contact quality or increase thermal load, pre-filtration becomes indispensable. However, guide design cannot rely solely on filtration: improving band guidance requires balancing the width of the guide jaws (to ensure film formation and particle tolerance) with the practical capability of the filtration system over the service life, given the evolving quantity and size of particles.

In addition to the effects of particulates, this study provides temperature-dependent viscosity data and classification of the cooling lubricant frictional behaviour relevant to sliding guidance. These data enable more accurate prediction of film thickness, heat generation, and vibration damping capacity in multi-parameter simulations of band–guide interaction at high-band speeds. Together, the results highlight three factors that are key to stable high-speed operation: (i) particle management through appropriate pre-filtration and maintenance intervals, (ii) gap design that can accommodate realistic debris while sustaining hydrodynamic/mixed lubrication, and (iii) the explicit consideration of temperature-dependent fluid properties in modelling and process setup.

The findings address notable gaps in the literature on band saws by quantifying in situ debris scales, linking them to guide clearances, and providing fluid and tribological parameters at operating temperatures. However, limitations include focusing on a specific emulsion and machine class, as well as the absence of long-term wear quantification. Future work should map particle concentration and size distributions to wear and thermal responses under controlled conditions, compare filter media and cut-off sizes with life-cycle costs, assess alternative guide materials and coatings, and extend modelling to gap-flow regimes with debris-laden and temperature-modified rheology. Overall, the present results provide a practical foundation for optimising cooling lubricant systems and guide geometries at higher band speeds, with the goal of extending tool life, stabilising guidance, and improving cut quality.

## Figures and Tables

**Table 1:** Cutting parameters.

Material	1.7225 (42CrMo4)
Cutting speed	$v_{c\;}$= 53 m/min
Feed rate	$f_{f}$= 26 mm/min
Emulsion	Avilub Metacool BFH; 12%
Cutting area	22.5 m^2^
Saw band guide clearance	0.1 mm

**Table 2:** Average steady-state friction coefficient of different media.

Medium	Steady-State Friction Coefficient µ (-)
Dry	3.130
Water	3.179
Cutting oil	0.104
Cooling lubricant	0.120

## Data Availability

The original contributions presented in this study are included in the article. Further inquiries can be directed to the corresponding author.

## Abbreviations

The following abbreviations are used in this manuscript:

HFRR	High-Frequency Reciprocating Rig
HSS	High-Speed Steel
MQL	Minimum Quantity Lubrication
MWF	Metalworking Fluid
EHL	Elastohydrodynamic films
